# Current State-of-the-Art Spectroscopic and Chromatographic Techniques Utilized in Food Authenticity and Food Traceability

**DOI:** 10.3390/foods13010003

**Published:** 2023-12-19

**Authors:** Giovanna Esposito, Marzia Pezzolato

**Affiliations:** Istituto Zooprofilattico Sperimentale del Piemonte, Liguria e Valle d’Aosta, Via Bologna 148, 10154 Turin, Italy; marzia.pezzolato@izsto.it

Food products are heterogeneous and complex matrices characterized by various compounds and in variable proportions. They can be of animal or vegetable origin and can come from different geographical areas, which can influence their composition. The globalization of supply chains, together with weak governance and the absence of standardized globally validated fraud prevention processes, increases the incidence of food irregularities.

Based on these considerations, the development of analytical methods to detect adulterations and verify the authenticity, geographical origin and traceability of foods is increasingly complicated.

Olive oil, fish, honey, milk and dairy products, meat, grain-based foods, wine, other alcoholic beverages and coffee are considered the matrices most at risk of fraud [1].

Nowadays, analytical approaches to food authentication and traceability are increasingly moving towards digital fingerprinting techniques. This Special Issue aims to fill the analytical gap in the search for food authenticity and to share new valid methods to control fraud in the food sector in order to protect consumers, food producers and the original product. Below are some examples of the most used techniques in this field.

Spectroscopic techniques, including vibrational near-infrared (NIR), mid-infrared (MIR), Raman, absorption ultraviolet-visible (UV-Vis), and nuclear magnetic resonance (NMR) are frequently used in combination with chemometric approaches to study the authentication and traceability of food.

Nuclear magnetic resonance (NMR), traditionally used for structural elucidation, has been used to authenticate olive oil and coffee [2,3,4].

Significant progress in spectroscopic research for the authentication of seafood has unfolded, particularly for fish mislabelling and substitution, such us the discrimination between fresh thawed fish [5,6], using NIR and NMR [5,6,7] or substitution detection with UV-Vis [8].

Chromatographic techniques combined with different mass spectrometry (MS) technologies [9], including time of flight spectrometry (TOF), high-resolution mass spectrometry (HRMS) and isotope ratio mass spectrometry (IRMS) [10], have been used extensively for food traceability and authentication, particularly for honey, wine, fish, coffee and rice [11,12,13].

Growing competition stemming from the globalization of markets calls for the development of highly innovative methods to test the authenticity and traceability of foods. This achievement would represent a milestone in ensuring label matching, detecting food fraud and avoiding scandals.
